# HDAC inhibition ameliorates cone survival in retinitis pigmentosa mice

**DOI:** 10.1038/s41418-020-00653-3

**Published:** 2020-11-06

**Authors:** Marijana Samardzija, Andrea Corna, Raquel Gomez-Sintes, Mohamed Ali Jarboui, Angela Armento, Jerome E. Roger, Eleni Petridou, Wadood Haq, Francois Paquet-Durand, Eberhart Zrenner, Pedro de la Villa, Günther Zeck, Christian Grimm, Patricia Boya, Marius Ueffing, Dragana Trifunović

**Affiliations:** 1 Lab for Retinal Cell Biology, Department of Ophthalmology, University of Zürich, Zürich, Switzerland; 2 Department of Neurophysics, NMI Natural and Medical Sciences Institute at the University of Tübingen, Reutlingen, Germany; 3 Institute for Ophthalmic Research, Eberhard Karls Universität Tübingen, Tübingen, Germany; 4 Graduate School of Neural Information Processing/International Max Planck Research School, Tübingen, Germany; 5 Department of Cellular and Molecular Biology, Centro de Investigaciones Biológicas Margarita Salas, CSIC, Madrid, Spain; 6 Paris-Saclay Institute of Neuroscience, CERTO-Retina France, CNRS, Univ Paris Sud, Université Paris-Saclay, Saint-Aubin, France; 7 Universidad de Alcala de Henares, Madrid, Spain; 8 Present Address: Institute of Neuronal Cell Biology, Technische Universität München, München, Germany

**Keywords:** Neuroscience, Neurological disorders

## Abstract

Cone photoreceptor cell death in inherited retinal diseases, such as Retinitis Pigmentosa (RP), leads to the loss of high acuity and color vision and, ultimately to blindness. In RP, a vast number of mutations perturb the structure and function of rod photoreceptors, while cones remain initially unaffected. Extensive rod loss in advanced stages of the disease triggers cone death by a mechanism that is still largely unknown. Here, we show that secondary cone cell death in animal models for RP is associated with increased activity of histone deacetylates (HDACs). A single intravitreal injection of an HDAC inhibitor at late stages of the disease, when the majority of rods have already degenerated, was sufficient to delay cone death and support long-term cone survival in two mouse models for RP, affected by mutations in the phosphodiesterase 6b gene. Moreover, the surviving cones remained light-sensitive, leading to an improvement in visual function. RNA-seq analysis of protected cones demonstrated that HDAC inhibition initiated multi-level protection via regulation of different pro-survival pathways, including MAPK, PI3K-Akt, and autophagy. This study suggests a unique opportunity for targeted pharmacological protection of secondary dying cones by HDAC inhibition and creates hope to maintain vision in RP patients even in advanced disease stages.

## Introduction

In Retinitis Pigmentosa (RP), the leading cause of inherited blindness, mutations in more than 90 genes affect the survival and/or function of rod photoreceptors or retinal pigment epithelium cells (RPE) (http://www.sph.uth.tmc.edu/Retnet/home.htm). One of the particularities of RP is that despite being mutation-unaffected, cone photoreceptors die secondarily once most rods are lost [1]. In humans, loss of rods initially has only minor consequences for vision, and the majority of patients are unaware of their condition until they start experiencing a prominent reduction in the central visual field, acuity, or color discrimination, due to the loss of cones. Hence, in a clinical setting, it is highly pertinent to develop therapies to treat advanced stages of RP, when the majority of rods have already degenerated, and cone degeneration has set in [2].

Despite the importance of cones for human vision, studies on therapeutic options to prevent their loss at advanced stages of RP are disproportionally low [1, 3–6] due to the intercellular relationship between rod and cone photoreceptors in human and mouse retina, where cones represent less than 5% of all photoreceptors [7]. Moreover, current knowledge suggests that the massive loss of rods in late RP creates a “point of no return”, after which cone cell death is unstoppable [2, 8], as cones are suffering from the loss of structural and nutritional support from rods [1, 9], exposure to oxidative stress [4], and inflammation [3, 6]. Although alleviating each of these processes individually has the potential to preserve cones to some extent [1, 3, 5, 10], an ideal therapeutic option should provide multi-level protection of cones in the rod-depleted retina. One way to achieve this could be by an epigenetically driven simultaneous regulation of several genes involved in diverse pro-survival responses.

Histone deacetylases (HDACs) are regulators of the chromatin structure, and changes in their activity affect transcription of a number of genes [11]. Tightly packed chromatin, following the HDAC-governed removal of acetyl groups from histones, is generally associated with transcriptional silencing, albeit this largely depends on the type and “health” status of cells [12]. Aberrant HDAC activity is causatively linked to various diseases ranging from cancer, to neurodegenerative diseases [12, 13]. We and others have previously shown that epigenetic regulation, via HDAC inhibition, can protect primary degenerating photoreceptors in inherited retinal dystrophies caused by mutations in different genes [14–17]. Consequently, more than 90 clinical trials involving HDAC regulators stress HDAC inhibition as a promising therapeutic approach for various diseases, including retinal dystrophies [12, 18].

Here, we investigated the involvement of HDACs in secondary cone degeneration in mouse models of RP. We found an increased HDAC activity present in both mutation-affected rods and in secondary dying cones. A single intravitreal injection of the HDAC inhibitor Trichostatin A (TSA) afforded long-term preservation of cone photoreceptors. Transcriptional changes associated with cone survival comprised regulation of distinct pro-survival mechanisms, including autophagy, MAPK, and PI3K/Akt regulation. Thus, therapies based on HDAC inhibition can offer a unique possibility to attenuate the loss of photoreceptors independent of the stage of degeneration.

## Materials and methods

### Animals

The C3H *rd1/rd1 (rd1)*, C57BL/6J x C3H *HR2.1:TN-XL x rd1 (rd1*^*TN-XL*^*)*, C57BL/6J *rd10/rd10 (rd10)*, and C57BL/6J wild-type (wt) mice were housed under standard light conditions, had free access to food and water, and were used irrespective of gender. *rd1*^*TN-XL*^ mice express the TN-XL (Ca^2+^ biosensor) selectively in cone photoreceptors under the control of the human red opsin promoter (HR2.1) [19, 20]. The presence of TN-XL biosensor does not alter the *rd1* phenotype, while it enables direct visualization of cone photoreceptors by fluorescence microscopy [20]. All procedures were performed in accordance with the ARVO statement for the Use of Animals in Ophthalmic and Vision Research, the regulations of the Tuebingen University committee on animal protection, Germany, veterinary authorities of Kanton Zurich, Switzerland and the ethics committees of the CSIC and the Comunidad de Madrid.

### Intravitreal injections

Single intravitreal injections were performed at postnatal day (PN) 19 in *rd1*^*TN-XL*^ and PN42 in *rd10* mice, as previously described [14]. Mice were anesthetized subcutaneously with a mixture of ketamine (85 mg/kg) and xylazine (4 mg/kg). One eye was injected with 0.5 µl of a 100 nM TSA (catalog T8552, Sigma-Aldrich, St. Louis, MO) in 0.0001% DMSO, while the contralateral eye was sham-injected with 0.0001% DMSO and served as a control. Assuming the intraocular volume of mouse eye to be 5 µl [21], this procedure resulted in a final intraocular concentration of 10 nM TSA. For the open field behavioral test, *rd10* littermates were TSA- or sham-injected bilaterally at PN42.

### Retinal explant cultures

Organotypic retinal cultures from *rd1*^*TN-XL*^ animals, including the retinal pigment epithelium (RPE) were prepared under sterile conditions as previously described [14, 15]. PN19 or PN21 *rd1*^*TN-XL*^ animals were sacrificed, the eyes enucleated and pretreated with 0.12% proteinase K (ICN Biomedicals Inc.) for 15 min at 37 °C in HBSS (Invitrogen Inc.). Proteinase K activity was blocked by the addition of 10% fetal bovine serum, followed by rinsing in HBSS. Next, the cornea, lens, sclera, and choroid were removed, while the RPE remained attached to the retina. The explant was cut into a clover-leaf shape and transferred to a culture membrane insert (Corning Life Sciences) with the RPE facing the membrane. The membrane inserts were placed into six-well culture plates with Neurobasal-A medium (catalog 10888022) supplemented with 2% B27 (catalog 0080085-SA), 1% N2 (catalog 17502048), and L-glutamine (0.8 mM, catalog 25030032) (all from Invitrogen Inc.), and incubated at 37 °C in a humidified 5% CO2 incubator. The culture medium was changed every 2 days during the 7 days culturing period. Retinal explants were treated with 10 nM TSA, 1 µM Panobinostat (catalog S1030, Selleckchem), 20 µM LY294002 (catalog S1105, Selleckchem), and 10 µM U0126-EtOH (catalog S1102, Selleckchem) diluted in Neurobasal-A culture medium. For the PI3K-Akt and MAPK inhibition experiments, cultures were treated with TSA, LY294002, U0126, TSA + LY294002, and TSA + U0126 only for 2 days followed by the culture medium without compounds for additional 5 days. For controls, the same amounts of DMSO were diluted in the culture medium. Culturing was stopped after 7 days by 2 h fixation in 4% PFA, cryoprotected with graded sucrose solutions containing 10, 20, and 30% sucrose and then embedded in tissue freezing medium (Leica Microsystems Nussloch GmbH).

### Quantification of cone survival

The quantification of cones was performed by manually counting the number of TN-XL labeled cones (using the Zen event counter) on at least two retinal cross-sections cut along the dorsoventral axis, at the level of the optic nerve. Retinal cross-sections were used to quantify cone photoreceptors as the TN-XL biosensor is present throughout the cone photoreceptor, except the outer segment (IS) [19]. The presence of the biosensor in the cell body, axon and IS, hampers a clear separation of individual cell bodies from the IS and/or axon on flat-mount preparations at the late stages of *rd1*^*TN-XL*^ degeneration, where cones align horizontally to the INL due to the lack of structural support from rods (SI Appendix, Fig. S1A.) Retinal cross-sections with labeled nuclei enabled distinction between different parts of the cones and facilitated the counting of their cell bodies. Colabeling of *rd1*^*TN-XL*^ cones with a cone-specific antibody, cone arrestin (CAR), showed full overlap between two fluorescent signals confirming the specificity of TN-XL labeling both in degenerating and protected cones (SI Appendix, Fig. S2). Cones were quantified on multiple images projection (MIP) obtained from 9–15 optical sections taken with 20x magnification at four positions in the retina: ventral and dorsal central retina (corresponding to −10° and 10° of eccentricity from the optic nerve, respectively) and ventral and dorsal peripheral positions at −80° and 80° degrees (SI Appendix, Fig. S1). For the quantification of cone survival in *rd10* retinas, cones were labeled with an antibody against cone arrestin. Spider diagrams show the number of cones per 100 µm of the outer nuclear layer (ONL) length at each position, presented as mean values ± SEM.

### Histology

For retinal cross-sectioning, the eyes were marked nasally, and eyecups (after cornea, iris, lens, and vitreous removal) were fixed in 4% paraformaldehyde for 2 h at room temperature. Following graded sucrose cryoprotection eyes were embedded in optimal cutting temperature compound (Tissue-Tek), cut into 12 μm sections, and mounted with Vectashield medium containing 4’,6-diamidino-2-phenylindole (DAPI, Vector). For retinal flat mounts, retinas without RPE were fixed for 30 min, cut into a cloverleaf shape, and mounted with Vectashield with the photoreceptors facing up. To analyze retinal morphology, eyes were fixed in 2.5% glutaraldehyde, cut at the optic nerve level, followed by 1% osmium tetroxide treatment post-fixation and ethanol dehydration, according to a previously described protocol [22]. After embedding in Epon 812, 0.5 μm thick sections were counterstained with toluidine blue. Immunostaining was performed on retinal cryosections by incubating with primary antibodies against rabbit cone arrestin (1:1000; catalog AB15282, Merck Chemicals GmbH), mouse anti-rhodopsin (1:400, catalog MAB 5316, Chemicon), LC3B (1:100, catalog NB-100-220, Novus) and LAMP1 (1:100, clone 1D4B, DSHB) at 4 °C overnight. Alexa Fluor 488, 568, or 647-conjugated antibodies were used as secondary antibodies. Images were captured using Z-stacks on a Zeiss Axio Imager Z1 ApoTome Microscope using 20x air, 40x oil, or 100x oil objectives. For the quantification of the LC3 and LAMP1 puncta in cones, 4 images per retina for in vivo treatment and 8 images per explant were assessed in each confocal plane obtained by the Leica TCS SP5 Confocal Microscope. Colocalizing puncta were counted using the counter plugin of Image J. The number of colocalizing puncta was divided by the number of cones in the whole z-stack.

### HDAC in situ activity assay

HDAC activity assays were performed on 12 µm thick cryosections of 4% PFA- fixed eyes following immunostaining against cone arrestin/rhodopsin as previously described [14]. Briefly, retina sections were exposed to 200 μM Fluor de Lys-SIRT2 deacetylase substrate (Biomol) with 500 μM NAD + (Biomol) in assay buffer (50 mM Tris/HCl, 137 mM NaCl; 2.7 mM KCl; 1 mM MgCl2; pH 8.0) for 3 h at room temperature. Following methanol fixation at -20 °C for 20 min, a developer solution (1x Biomol; KI105) containing 2 μM TSA and 2 mM nicotinamide in assay buffer was applied to generate the signal. Due to the presence of a background staining in negative controls, only cells with prominent nuclear staining were considered as HDAC positive [14].

### MEA recording

Retina explant cultures attached to the membrane were transferred from the incubation chamber to a 256-electrode MEA (Multi channel systems MCS GmbH, Reutlingen, Germany) with the ganglion cell side facing the electrodes. A custom-made grid was placed over the retina to improve the contact between electrodes and the tissue and the stability of the recording. Cultures were perfused throughout the experiment with oxygenated Ames’ medium (A1420, Sigma-Aldrich) and heated to 36 ˚C. The electrode spacing was 200 µm, with the total recordings area of ~3.2 × 3.2 mm^2^. Twenty repetitions of 350 ms long light-flashes of increasing intensity (8.6 e12, 5.3 e13, 3 e14, 8.7 e14, 1.6 e15, 2.3 e15 photons/cm^2^ s) separated by 2 s of dark were presented to both control and the TSA-treated retinal explants mounted on MEA. The recordings were made with a sampling rate of 25 kHz using the MC Rack software (Multi Channel Systems MCS GmbH). The analysis of recordings from 256-MEAs was performed using Python 3.6. Recordings were bandpass - filtered (400–5000 Hz, Butterworth 2nd order), and spikes were detected as threshold crossing of 5 times the standard deviation of the filtered signal with a pause time of 1.5 ms. Light-induced RGCs activation recorded by an electrode was quantified for each light intensity using a two-tailed, paired *t*-test comparing the detected spikes during the 20 repetitions of light-ON (350 ms of light flash) or light-OFF (350 ms after the light shut-off) versus the spontaneous activity recorded before light onset. Only electrodes with a statistically significant difference (*p* < 0.01 and t-statistic > 2) were considered light-activated. In addition, only channels light-activated for at least 3 out of 5 light intensities in the photopic light range (5.3 e13, 3 e14, 8.7 e14, 1.6 e15, 2.3 e15 photons/cm^2^) were included in the analysis. These criteria were used to eliminate potential non-stable recordings. To quantify the degree by which light onset changes the spontaneous firing rate and to avoid an overestimation of the electrodes recording low spontaneous activity, a response ratio was calculated. The calculation is based on the well-established bias index used to quantify physiological light responses [23, 24] and is analogous to the Michelson contrast used to quantify the contrast in visual images:

Response ratio = (Firing rate LIGHT ON – Firing rate spontaneous)/(Firing rate LIGHT ON + Firing rate spontaneous).

### Open field behavioral test

The open field test is based on natural mouse behavior to avoid brightly lit open spaces [25]. This test was performed based on the previously described procedure with minor modifications [26]. A custom-made box for light/dark transition test (60 × 30 × 30 cm) was divided into two chambers, one black and one white, connected by a door (5 × 5 cm). The mice were habituated in the dark for at least 1 h before the testing. The light chamber was illuminated from the top by white diodes (670 lux, LitePad® HO90, Rosco). Mice were placed into the dark compartment, and the door was opened after 3 s. Mice were allowed to move freely between the two chambers with the door open for 5 min. The time spent in the dark chamber was registered by an experimenter. After each trial, all chambers were disinfected to prevent a bias based on olfactory cues.

### ERG recordings

Mice were dark adapted overnight, and subsequent manipulations were performed in dim red light. Mice were anesthetized with intraperitoneal injections of ketamine (95 mg/kg) and xylazine (5 mg/kg) solution and maintained on a heating pad at 37 °C. Pupils were dilated with a drop of 1% tropicamide. To optimize electrical recording, a topical drop (2% Methocel) was applied to each eye immediately before placing the corneal electrode. Flash-induced ERG responses were recorded from the right eye in response to light stimuli produced with a Ganzfeld stimulator. Light intensity was measured with a photometer at the level of the eye (Mavo Monitor USB). Four to 64 consecutive stimuli were averaged with an interval between light flashes in scotopic conditions of 10 s for dim flashes and up to 60 s for the highest intensity. Under photopic conditions, the interval between light flashes was fixed at 1 s. ERG signals were amplified and band-filtered between 0.3 and 1000 Hz with an amplifier (CP511 AC amplifier). Electrical signals were digitized at 20 kHz with a power laboratory data acquisition board (AD Instruments). Under dark adaptation, scotopic threshold responses (STR) were recorded to light flashes of −4 log cd·s·m^−2^; rod responses were recorded to light flashes of −2 cd·s·m^−2^, and mixed responses were recorded in response to light flashes of 1.5 log cd·s·m^−2^. Oscillatory potential (OP) was isolated using white flashes of 1.5 log cd·s·m^−2^ in a recording frequency range of 100–10,000 Hz. Under light adaptation, cone-mediated responses to light flashes of 2 log cd·s·m^−2^ on a rod-saturating background of 30 cd·m^−2^ were recorded. Wave amplitudes of the STR, rod responses (b-rod), mixed responses (a-mixed and b-mixed), and OP were measured offline by an observer masked to the experimental condition of the animal.

### Flow-sorting of the cone photoreceptors

A protocol from Palfi et al. [27]. was used to dissociate retinal cells. Briefly, PN19-26 control and TSA-treated retinal explant cultures were removed from membranes and incubated in trypsin (Sigma-Aldrich) solution for 20 min at 37 °C. Following incubation with trypsin inhibitor (Sigma-Aldrich), cell suspension was washed with HBSS and passed through a 40-µm filter before fluorescence activated sorting (FACS). One biological replicate included at least 2 retinal explants prepared from different animals. Three independent biological replicates from control and TSA-treated retinal explants were used for cone photoreceptor FACS using an ARIAIII cell sorter (BD Biosciences). The sort was performed with a 100 µm nozzle tip, at a sheath pressure of 20.0 psi, and with purity precision. TN-XL positive cone photoreceptors were gated as follows: singlets forward scatter (FSC-A vs FSC-H)/singlets side scatter (SSC-A *vs*. SSC-H)/viable cells (FSC-A *vs*. SSC-A)/TN-XL + cells (FSC-A *vs*. TN-XL-A) (gating Fig. S5A, B). The purity of sorted TN-XL + cells was checked by performing post-sort FACS analysis (Fig. S5C, D).

### Whole transcriptome sequencing and data analysis

2000–5000 frozen sorted-cells were lysed in ~10 µl of lysis buffer, and cDNA synthesis was performed using the SMART-Seq v4 Ultra Low Input RNA Kit (catalog 634888, Takara Bio). First-strand cDNA synthesis was performed using 20–50% of the input, and was followed by full-length double-strand cDNA amplification using 17 PCR cycles. The quality of the resulting cDNA was validated using Bioanalyzer and High Sensitivity DNA Kit (Agilent), and Qubit dsDNA HS fluorometric quantification (ThermoFisher Scientific). NGS libraries were prepared using 150 pg of cDNA input and the Nextera XT DNA Library Preparation Kit (catalog FC-131-1024, Illumina) with 11 cycles of PCR. Libraries were sequenced as single reads (75 bp read length) on a NextSeq500 (Illumina) with a depth of >20 million reads. Library preparation and sequencing procedures were performed by the same individual, and a design to minimize technical batch effects. Data quality of raw RNA-seq reads in FASTQ files was assessed using ReadQC (ngs-bits version 2018_04) to identify potential sequencing cycles with low average quality and base distribution bias. Reads were preprocessed with Skewer (version 0.2.2) and aligned using STAR (version 2.5.4a), allowing spliced read alignment to the mouse reference genome (Ensembl Mus musculus GRCm38). Alignment quality was analyzed using MappingQC (ngs-bits version 2018_04) and visually inspected with Broad Integrative Genome Viewer (version 2.4.0). Based on the genome annotation ITAG (Ensembl 75), normalized read counts for all genes were obtained using subread (version 1.6.0) and edgeR (version 3.28.0). Raw counts data were processed using iDEP, an integrated web application for RNA-seq data analysis [28]. To provide access to RNAseq data, we generated a BioJupies notebook [29] link providing an interactive and visual analysis of all the data (https://amp.pharm.mssm.edu/biojupies/notebook/LjknTl51J). Sequencing data are deposited on GEO with the accession number GSE141601.

For differential gene expression (DEG) analysis, gene counts were filtered to only retain genes with a value > 1 cpm (count per million), in at least two samples within at least one group (control or treated), leaving around 14,400 genes for determination of differential expression in each of the pair-wise comparisons between experimental groups. Differentially expressed genes between treated and control groups were identified using the two-tailed permutation FDR-based Student’s *t* test (FDR < 0.05 and 250 randomizations). Only transcripts coding for protein sequences were retained for pathway analysis. Quantitative gene expression data was submitted and integrated into the PaintOmics 3 data analysis platform [30, 31], in order to identify trends in pathway modulation following the TSA treatment. A stable link is provided to access and visualize the results (http://www.paintomics.org/?jobID=4CIbSr1eA4).

### Quantitative RT-PCR

For qRT-PCR, 2000–6000 cones from two pooled PN19-26 ex vivo *rd1*^*TN-XL*^ explants were sorted in 350 µl RLT buffer (Qiagen) to lyse cells. RNA extractions were performed using an RNeasy Micro Kit (Qiagen), followed by cDNA synthesis using the QuantiTect Reverse Transcription Kit (Qiagen). qRT-PCR reactions were performed in technical duplicates of 4 biological samples of TSA-treated and control retinas, on BioRad CFX96 real-time system using QuantiTect SYBR Green PCR Kit (Qiagen) along with gene specific forward (fwd) and reverse (rev) primers (250 nM). The sequences of the primer sets used are listed in supporting Table S1. The PCR protocol included 40 cycles of: 94 °C (15 s), 57 °C (30 s), and 72 °C (30 s). Relative mRNA expression of each gene of interest was calculated using ∆∆CT method and GAPDH as a housekeeping gene.

### Statistics

All data were analyzed using Excel (Microsoft) and GraphPad Prism 6. For each comparison between control and treated groups, normal distribution was determined with GraphPad software (D’Agostino & Pearson omnibus and Shapiro–Wilk normality tests). When applicable (methodology based) investigators were blinded for the condition. Quantification of in vivo survival for every time points, for different positions, was calculated using two-way ANOVA. The overall temporal survival curve shown in Fig. 2R was obtained by averaging all the analyzed positions from all the animals per stage. The statistical significance in the broadening of the survival curves was assessed *via* shared control Cummings estimation plot using the DABEST package in R with 5000 bootstraps resamples (https://www.estimationstats.com/) [32]. A shared-control estimation plot represents the Hedges’ *g* comparison of log2-transformed cone survival ratios. Since TSA treatment prevented *rd1*^*TN-XL*^ cone loss up to 7 days post-injection, the cone survival ratio at PN26 was used as the control and compared to the survival ratio at PN30, PN37, PN45, PN60 and PN90. The statistical significance of Hedges’ *g* difference was determined by unpaired two-sided permutation *t*-test.

Ex vivo survival was quantified on 5–12 different positions, from 3–5 retinal cross-sections obtained from different positions within a retinal explant. Unpaired, two-tailed *t*-tests were used to compare controls with treatments.

Statistical differences for LC3-LAMP1 puncta quantification and qRT-PCR were calculated using Mann–Whitney nonparametric test.

RNAseq raw data were processed and normalized using subread (version 1.6.0) and edgeR (version 3.28.0). Statistical data analysis was carried out using the Perseus tool suite for Omics data analysis [33]. Two-tailed, unpaired permutation-based FDR Students’ *t* test on biological replicates’ mean difference was applied (FDR < 0.05 and 250 randomizations). Log2 fold changes, mean differences, and *p* values are reported in the Gene expression dataset.

The statistical difference of light-induced response ratio between control and treated retinas was calculated using a Wilcoxon rank-sum test.

Statistical difference of open field behavioral test was calculated using a two-way ANOVA test.

## Results

### Secondary cone degeneration in RP is associated with an increased HDAC activity

**Fig. 1:**
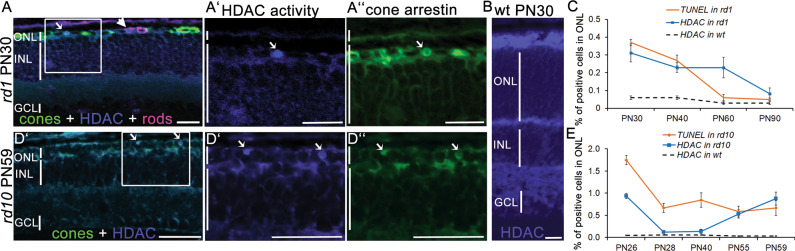
Increased HDAC activity in RP photoreceptors at advanced stages of degeneration. **A**–**A**” HDAC in situ activity assay in *rd1* retinal cross-sections at PN30. HDAC activity (blue) was present in both rod (magenta, arrowhead, rhodopsin immunostaining) and cone photoreceptors (green, arrow, cone arrestin immunostaining). **A**’ Magnification of the marked region indicating HDAC+ cell colocalizing with cone arrestin (**A**”). **B** HDAC activity in wild-type retina at PN30. **C** Percentage of TUNEL+ and HDAC+ cells in the ONL of *rd1* and wt mice over time. **D**–**D**” Representative image of a colocalization of HDAC activity and cone arrestin in *rd10* mice at PN59, with only cones remaining in the ONL. **E** Percentage of TUNEL+ and HDAC+ cells in the ONL of *rd10* and wt mice over time. Data are shown as mean ± SEM (*n* = 3–5 animals per age). Scale bars: 50 µm. ONL outer nuclear layer, INL inner nuclear layer, GCL ganglion cell layer, PN post-natal day.

To assess the involvement of HDACs in the secondary cone degeneration, we used an HDAC in situ activity assay [16] at advanced stages of photoreceptor degeneration in two mouse models for RP, the *rd1* and *rd10* mice. Different mutations in the rod-specific phosphodiesterase 6b (*Pde6b*) gene lead to fast rod photoreceptor degeneration, with the onset at PN9 in *rd1* and PN14 in *rd10* mice [15, 34, 35]. Cone degeneration begins once most rods have degenerated, around PN20 in the *rd1* and PN40 in the *rd10* mouse [1, 3]. At PN30, the outer nuclear layer (ONL) in *rd1* mice is reduced to only one row of photoreceptors, almost exclusively cones (Fig. 1A).

A combination of in situ HDAC activity assay with cone- and rod-specific immunostaining confirmed previously established association of increased HDAC activity and rod degeneration (Fig. 1A) [15, 16]. Interestingly, HDAC activity also colocalized with the cones, once the majority of rods have degenerated (Fig. 1A–A”) [1]. The presence of HDAC positive cells within the ONL was detected through the entire period of cone cell death in *rd1* mice (Fig. 1C), while no positive signal for HDAC activity was detected in nuclei of wild-type mice at PN30 (Fig. 1B). Moreover, late stages of *rd10* degeneration also displayed increased HDAC activity, mirroring the timeline of photoreceptor cell death in the *rd10* mouse, characterized by massive rod loss up to PN26 [35], followed by continuous photoreceptor cell death, likely reflecting the simultaneous rod and cone loss (Fig. 1D, E). These findings suggest that the cell death of both mutation-affected rods and secondary dying cones may be associated with HDAC overactivation.

### HDAC inhibition prolongs cone survival in RP mice

**Fig. 2:**
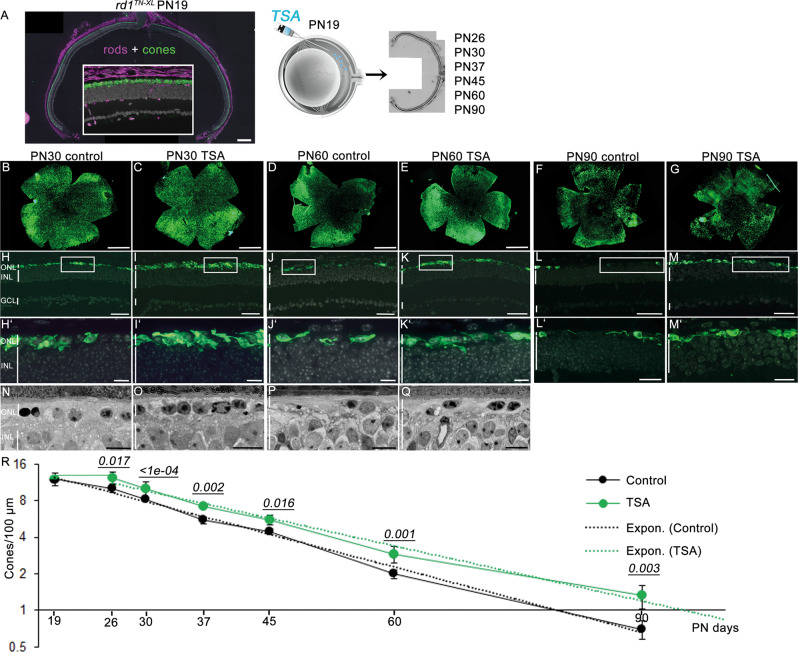
HDAC inhibition promotes long-term cone survival in *rd1*^*TN-XL*^ mice. **A** Retinal cross-section of an *rd1*^*TN-XL*^ mouse at PN19, showing TN-XL-labeled cones (green), rods (magenta, anti-rhodopsin antibody), and nuclei (gray, DAPI). Note that the mouse secondary antibody used to detect anti-rhodopsin antibodies showed the non-specific signal in layers other than ONL. Schematic representation of a single intravitreal injection of TSA at PN19, followed by quantification of cone survival up to PN90. **B**–**G** Flat mounted retinas from control and TSA-treated eyes at indicated PN days. **H**–**M** Representative images of retinal cross-sections from control and TSA-treated retinas at PN30, PN60 and PN90 used to quantify of TN-XL labeled cones. **H**’–**M**’ Magnifications of the marked regions shown in **H**–**M**. **N**–**Q** Retinal morphology of control and treated retinas at PN30 and PN60. **R** Quantification of cone survival in control and TSA-treated retinas. *Y* axis is in log2 scale. Data are shown as means ± SEM (*n* = 5–10 animals per age). Numerical *p* values by two-way ANOVA. The fitting of the exponential curves is shown in dotted lines. Scale bars: **A**, **H**–**M** 50 µm; **B**–**G** 500 µm; **H**’–**M**’20 µm; **N**–**Q** 10 µm. ONL outer nuclear layer, INL inner nuclear layer, GCL ganglion cell layer, PN postnatal days.

To determine if HDAC inhibition has the potential to prevent secondary cone degeneration, we injected a well-established class I and II HDAC inhibitor, TSA, in the *rd1*^*TN-XL*^ mouse that expresses the fluorescent TN-XL biosensor exclusively in M- and L-cones [19] (Figs. S1, S2). To minimize the indirect positive effects of increased rod survival afforded by HDAC inhibition on the *rd1* cone survival, we selected a late time-point to start the treatment, PN19. Already from PN19, the *rd1*^*TN-XL*^ ONL is reduced to only one row of photoreceptors, almost exclusively cones (Fig. 2A). After a single intravitreal injection of TSA at PN19, we assessed cone survival up to 3 months of age at different time points: PN26, PN30, PN37, PN45, PN60, and PN90. Whole-mount preparations of sham-injected eyes showed center to periphery gradient of cone loss at PN30 (Fig. 2B). In contrast, an increased cone-specific fluorescent signal was detected in treated contralateral eyes (Fig. 2C). While at PN60 and PN90, loss of cones had proceeded in control eyes, with cones remaining only at the far periphery (Fig. 2D, F), the treated eyes showed higher immunofluorescent signals, suggesting enhanced cone preservation in the central retina up to 3 months of age (Fig. 2E, G). An increase in cone survival was even more evident on retinal cross-sections, where individual cone cell bodies and remaining cone segments could be clearly identified (Figs. 2H–M’, S1, S2). A higher number of cones in TSA-treated animals was also evident in retinal light micrographs (Fig. 2N–Q). While untreated retinas showed a reduced density of nuclei, many of which were pyknotic, the treated retina displayed healthier cone morphology with classical heterochromatin distribution (Fig. 2O, Q) [22]. A single intravitreal injection was sufficient to prevent loss of *rd1*^*TN-XL*^ cones for up to 7 days, while in control retinas, cone degeneration continued with ~15% fewer cones than at the beginning of the treatment. A plot of cone numbers up to PN90 showed that cone loss in control and treated retinas followed an exponential decay function, with the two curves clearly separated (Fig. 2R). In addition, fitted trend lines showed significantly broader separation of the two curves at PN90 (Figs. 2R and S3) and predicted an X-axis intersection of the treated curve with a delay of 16 days, compared to control (PN79 for treated *vs*. PN95 for control retinas, Fig. 2R). These extrapolations suggest that the TSA treatment not only delayed but also slowed down secondary cone degeneration.

**Fig. 3:**
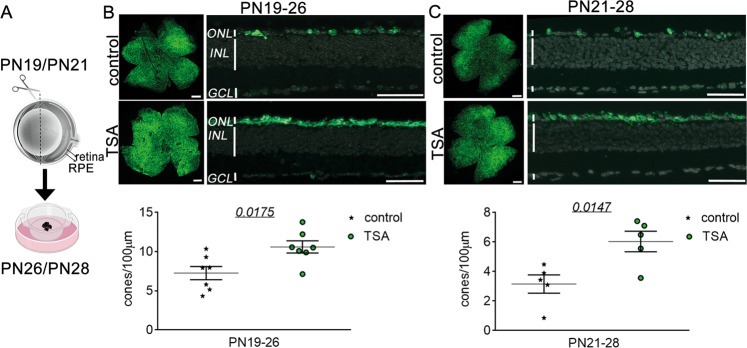
HDAC inhibition protects *rd1*^*TN-XL*^ cones ex vivo. **A** Schematic representation of the ex vivo retinal explants preparation. Retinas from *rd1*^*TN-XL*^ mice were collected at PN19 or PN21 and treated for 7 days with control or TSA-medium. **B**–**C** Representative flat mount preparations and cross-sections of explanted retinas, that were used for the quantification of cone survival, shown in the dot plot below. Data are shown as mean ± SEM (*n* = 5–7 animals per age group). Numerical *p* values by unpaired, two-tailed *t*-test. Scale bars in whole mounts 500 µm; retinal cross-sections 50 µm. RPE retinal pigment epithelium, ONL outer nuclear layer, INL inner nuclear layer, GCL ganglion cell layer, PN postnatal day.

To analyze the effects of continuous TSA treatment on cone survival, we treated *rd1*^*TN-XL*^ retinal explants from PN19 until PN26 ex vivo. Retinal cultures, consisting of the retina and RPE layer, enable maintaining mature neurons in situ, and complex neuronal connections while providing the possibility for constant exposure to a drug via a culture medium (Fig. 3A). Similarly to *rd1*^*TN-XL*^ cone degeneration in vivo, the center to periphery gradient of cone loss was more prominent in the control explant cultures (Fig. 3B). Quantification of cone numbers in retinal cross-sections showed ~30% increase in the cone survival in TSA-treated retinal explants (Fig. 3B).

Next, we asked if the HDAC inhibition has the potential to protect secondary dying cones even at a later stage of degeneration. For this, we started the treatment at PN21 and assessed the *rd1*^*TN-XL*^ cone survival after one week in culture (Fig. 3C). Also in this case, the TSA treatment significantly improved cone survival, with 52% more cones in the treated explants. We also tested the neuroprotective properties of Panobinostat, a clinically approved pan-HDAC inhibitor within the same group of inhibitors as TSA [36]. As with TSA, 7 days treatment with Panobinostat increased *rd1*^*TN-XL*^ cone survival up to 30% (Fig. S4).

### HDAC inhibition improves cone-mediated light-responses

**Fig. 4:**
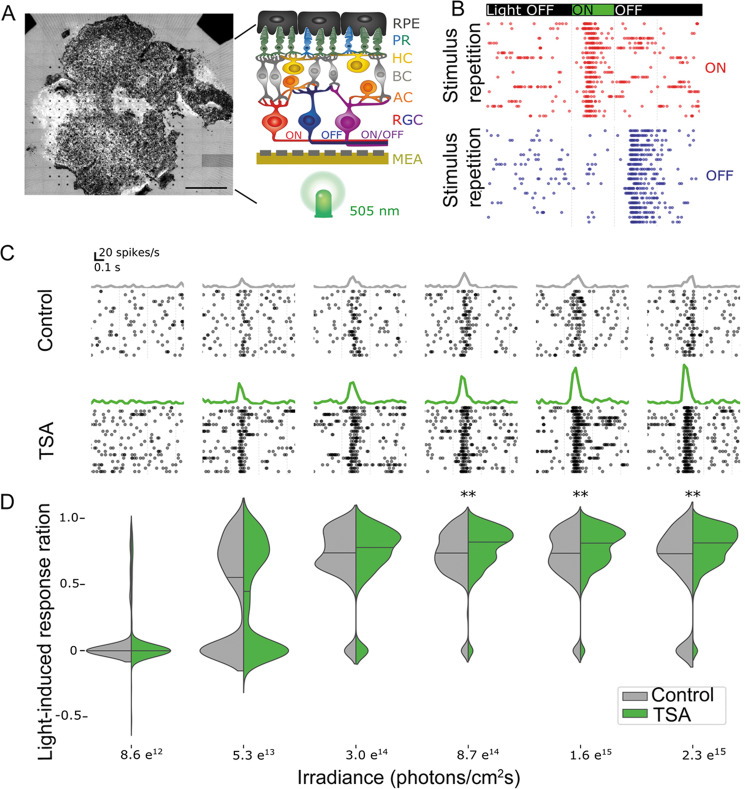
HDAC inhibition improves cone function in *rd1*^*TN-XL*^ retinal explants. **A** A micro-electrode array (MEA) with 256 electrodes was used to record retinal ganglion cell (RGC) responses in control and TSA-treated PN19-26 *rd1*^*TN-XL*^ retinal explants. Schematic drawing of the MEA setup used in the study. **B** Representative recordings of ON and OFF responses from TSA-treated retinal explants during stimulation with flickering light (350 ms flashes, 505 nm) shown as raster plots. Each dot indicates one spike. The green bar indicates the start of light stimulation. **C** Exemplary spike responses obtained in ontrol (gray) and treated (green) explants at six different light intensities, shown as raster plots (bottom) and averaged firing rate histograms (top). **D** Quantification and discrimination of the response ratio in control (gray) and treated explants (green). Significant differences based on Wilcoxon rank-sum test are detected for three high light intensities (*n* = 161 channels from eight control, *n* = 274 from nine treated retinas, **p < 0.01). RPE retinal pigment epithelium, PR photoreceptors, HC horizontal cells, BC bipolar cells, AC amacrine cells, RGC retinal ganglion cells.

We then wanted to see whether the remaining cones were light-sensitive and able to drive functional responses. We used a micro-electrode array (MEA) [37] to record the light‐mediated spiking activity of retinal ganglion cells (RGCs) of *rd1*^*TN-XL*^ retinal explants. The experimental setup included a light source at the bottom of the MEA chamber, mimicking the physiological situation where light stimulation comes from the ganglion cell side, while the RPE layer on the top of explants provided a physiological environment for light absorption (Fig. 4A).

A LED emitting green light (505 nm) with increasing light intensities, far below the safety limit for the human eye [38], was used to stimulate cones in control and TSA-treated PN19-26 retinal explants. Light-ON and Light-OFF responses were detected in both control and treated retinas suggesting preservation of physiological network functionality (Fig. 4B). To estimate the degree of activity change upon light stimulation, we calculated a light-induced response ratio for ON responsive MEA electrodes. The light-induced response ratio quantifies how much the firing rate (Fig. 4C) increases during light stimulation and can be considered a simple link between the number of rescued cones and functional readout. The distribution of the light-induced response ratio, presented as a violin plot, demonstrates that increasing light intensities lead to a higher firing rate (Fig. 4D). Changes of the spontaneous activity under the same light intensities were not detected. For each condition (control *vs*. treatment), the light-induced response ratio of the two distributions were compared. While for low intensities, the median values in control and treated retinas were not significantly different, the light-induced response ratio increased in the treated samples and was significantly different above a light intensity of 8.7 e^14^ photons/cm^2^ sec (*p* < 0.01). The median light-induced response ratio in treated retinas reached 0.82 while in control condition it approached 0.74 (Fig. 4D).

**Fig. 5:**
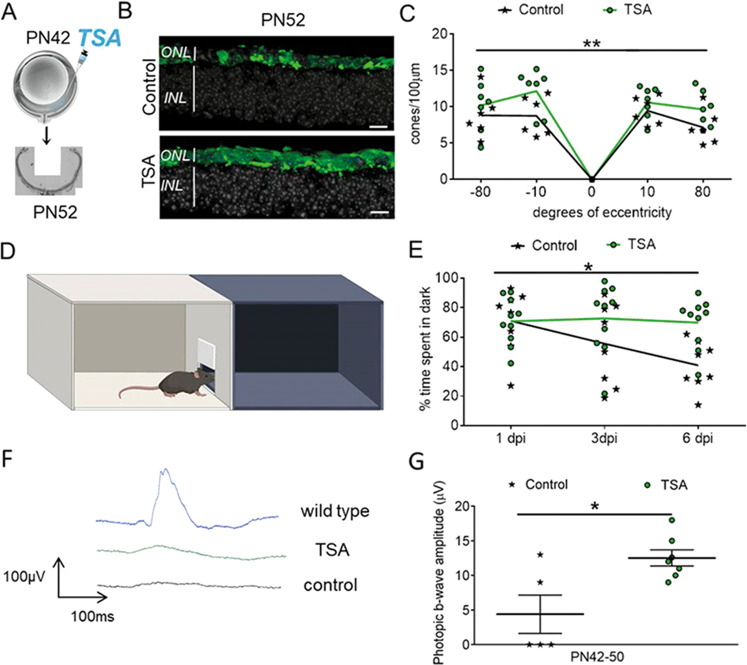
HDAC inhibition promotes cone survival and visual function in *rd10* mice. **A** A schematic representation of a single intravitreal TSA injection at PN42 followed by quantification of cones 10 days post-injection. **B** Representative images of retinal cross-sections from control and TSA-treated retinas at PN52 used to count the number of cones (cone arrestin-labeled cones in green, DAPI-labeled nuclei in gray). **C** Spider diagram showing the number of cones along the dorso-ventral axis, corresponding to 10°, 80°, −10°, and −80° of eccentricity from the optic nerve, respectively. **D** Drawing of the open filed behavioral test used to assess cone-mediated vision. **E** Percentage of time spent in the dark compartment for control and TSA-treated animals 1, 3, and 6 days post-injection (dpi). **F** Representative traces of photopic ERG recordings used to evaluate cone function in wild-type, TSA- and sham-treated *rd10* retinas at PN50. **G** Photopic b-wave amplitudes of control and TSA-treated *rd10* mice. Data are shown as means ± SEM (*n* = 5–10 animals per group). Statistical significance was assessed using two-way ANOVA (**C** and **E**) and Mann–Whitney nonparametric test (**G**), with *p < 0.05, **p < 0.01. Scale bars: 10 µm. ONL outer nuclear layer, INL inner nuclear layer, PN postnatal day.

To determine if HDAC inhibition has the potential to delay secondary cone death and preserve visual function independent of the underlying mutation, we injected *rd10* mice at a stage when the majority of rods have degenerated [1, 3]. A single intravitreal injection at PN42 resulted in a higher number of cones in TSA-treated compared to sham-treated retinas (Fig. 5A–C). Increased cone survival was accompanied by amelioration of vision-mediated behavior of *rd10* animals injected bilaterally with TSA (Fig. 5D, E). An improvement in cone function was also detected by electroretinography (ERG). At PN50, a modest but significant increase in photopic b-wave amplitudes was detected in TSA-treated retinas (Fig. 5F, G). Collectively our data suggest that HDAC inhibition has the potential to support cone survival and function at late stages of photoreceptor degeneration in different RP mouse models.

### HDAC inhibition induces global changes in gene-transcription patterns in the surviving cones

**Fig. 6:**
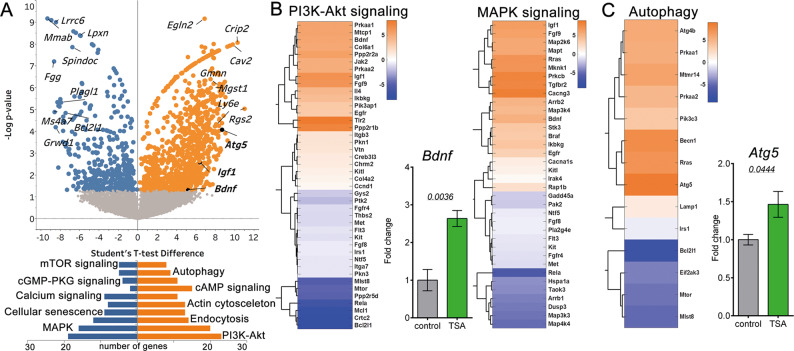
Whole genome transcriptomic analysis of TSA-induced survival of *rd1*^*TN-XL*^ cones. **A** A volcano plot representation of differentially expressed genes as detected by RNA-seq in flow-sorted cones from treated *vs*. untreated PN19-26 *rd1*^*TN-XL*^ (*n* = 3 animals). Orange and blue dots: significantly enriched and perturbed genes, respectively, in TSA-treated cones (FDR-based, Student’s *t* test of mean difference). PaintOmics analysis was used to identify differentially regulated pathways in TSA-protected cones. **B**, **C** Heat maps with hierarchical clustering of differentially expressed genes within PI3k-Akt, MAPK, and autophagy pathways. Genes showing Student’s *t* test difference between TSA and control, with *p* ≤ 0.05 were selected. Bar graphs: qRT-PCR validation of differential expression of *Bdnf* (**B**) and *Atg5* (**C**) in FACS-sorted cones. Fold changes are relative to controls. Data are shown as mean ± SEM (*n* = 4 animals). Numerical *p* values by Mann–Whitney nonparametric test.

We performed whole transcriptome analysis of flow-sorted *rd1*^*TN-XL*^ cones from retinal explants after seven days in culture (PN19-26). TN-XL-positive cells represented 95% of FASC-sorted cells, suggesting a highly purified cone population (SI Appendix, Fig. S5). RNA-seq analysis of differentially expressed genes (DEG) in TSA-treated *vs*. untreated cones revealed 1845 genes with significantly different expression (enrichment of 1163 and reduction in 682 genes), as indicated in the volcano plot (Fig. 6A, Supplementary gene expression dataset). To identify signature trends for activation or inhibition of downstream pathways, we used protein-coding genes with significant differential expression following the TSA treatment (*p* ≤ *0.05*), regardless of their fold change. Top molecular pathways differentially regulated following the TSA treatment included transcriptional changes of around 40 genes belonging to PI3K-Akt and/or MAPK signaling pathways and more than 20 genes regulating cellular senescence, endocytosis, actin cytoskeleton, as well as cAMP and calcium signaling (Fig. 6A, http://www.paintomics.org/?jobID=4CIbSr1eA4).

**Fig. 7:**
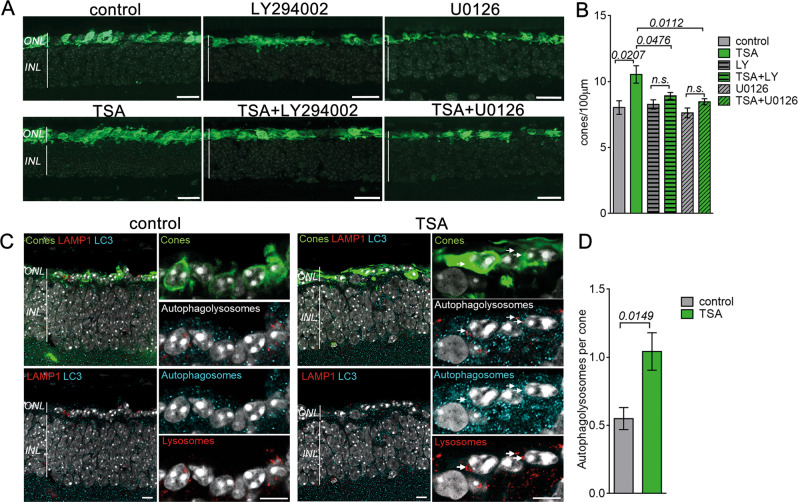
TSA-induced cone survival is modulated by PI3K-Akt, MAPK, and autophagy. **A** Retinal cross-sections of control (top) or TSA-treated (bottom) *rd1*^*TN-XL*^ retinas at PN26 incubated from PN19-PN21 with LY294002 or U0126. TN-XL cones appear green. **B** Quantification of cones in the ex vivo retinas at PN26. **C** Representative images from a single confocal plane on one retinal cross-section from an *rd1*^*TN-XL*^ mouse at PN26, sham- or TSA-injected at PN19, with TN-XL cones (green), autophagosomes (cyan, LC3B antibody), and lysosomes (red, LAMP1 antibody). The colocalization of autophagosomes and lysosomes is marked with arrows. **D** Quantification of the number of colocalized LC3-LAMP1 (autophagolysosomes) puncta per cone in the ONL. Data are shown as mean ± SEM based on 6–10 positions within retina, *n* = 6–8 animals in **B**; *n* = 3 animals in **D**. Numerical *p* values by Mann–Whitney nonparametric test. Scale bars in **A** 20 μm, in **C** 10 μm. ONL outer nuclear layer, INL inner nuclear layer.

The heatmap representation of the genes showing differential expression within the PI3K-Akt cascade (mmu04151) demonstrated the enrichment of about half of the genes in the TSA-treated cones (23/42 genes) (Fig. 6B). Similarly, 20 out of 36 genes were enriched within the MAPK cascade (mmu04010). Among highly abundant transcripts were the brain-derived neurotrophic factor (*Bdnf*), and the insulin-like growth factor 1 (*Igf1*), growth factors regulating MAPK and PI3K-Akt pathways. A significantly increased *Bdnf* expression in the TSA-treated cones sorted from independent samples was confirmed by quantitative PCR (qRT-PCR), while a tendency towards increased expression was observed for *Igf1* (Figs. 6B, S6). To determine if the survival of the TSA-treated cones was mediated *via* PI3K-Akt and/or MAPK pathways, we assessed cone survival in the *rd1*^*TN-XL*^ explants following the combined treatment with TSA and well-established inhibitors of PI3K-Akt and MAPK pathways, LY294002 and U0126, respectively. U0126 blocks MAPK signaling by inhibiting MEK1/2 [39], while LY294002 inhibits both the PI3K-Akt pathway and autophagosome formation [40, 41]. To avoid potential cytotoxic effects of prolonged treatment with PI3K-Akt and MAPK inhibitors, we assessed cone survival in *rd1*^*TN-XL*^ explants after one week in culture, but with inhibitors present only during the first 2 days. Interestingly, the TSA treatment of only 2 days afforded the same extent of cone protection as the 7 days treatment. Whereas LY204002 or U0126 had no effect on cone survival in control retinas, both inhibitors significantly decreased TSA-induced cone survival (Fig. 7A, B).

Pathway analysis also showed significant upregulation of 9/14 genes related to autophagy (mmu04140), an additional pro-survival mechanism (Fig. 6C) [42, 43]. Significant upregulation of the autophagy-related-5 gene (*Atg5*) in surviving cones was confirmed by qRT-PCR (Figs. 6C, S6). We also assessed the formation of autophagolysosomes in surviving cones by quantifying the colocalized puncta between an autophagosome marker, the microtubule-associated proteins 1A/1B light chain 3B (LC3B), and lysosome-associated membrane protein 1 (LAMP1) in control and treated *rd1*^*TN-XL*^ cones. LC3-LAMP1 staining of retinal cross-sections from PN19-26 in vivo TSA-treated mice showed an overall increase in both LC3- and LAMP1-positive vesicles, as well in colocalizing puncta within the cones in comparison to sham-treated retinas (Fig. 7C, D). Similarly, increased LC3-LAMP1 colocalization in cones was also detected in ex vivo treated *rd1*^*TN-XL*^ explants (Fig. S7).

Finally, to identify similar molecular signatures to the expression pattern obtained in this study, we submitted our data to the Library of Integrated Network-Based Cellular Signatures project (LINCS) (Fig. S8) [44]. The analysis matched our data to gene expression profiles of TSA and vorinostat treatments on human cell lines deposited in the LINCS platform. Of note, vorinostat is a clinically approved pan-HDAC inhibitor from the same class as TSA. This suggests a possibility for repurposing “off the shelf” HDAC-inhibiting drugs for the treatment of RP.

## Discussion

Cone photoreceptors are indispensable for human vision in daylight, and their loss has devastating effects on daily tasks and life quality [45]. Here, we show that cones have an innate ability for survival even in the total absence of rods, which can be induced by HDAC-driven transcriptional changes. At the same time, HDAC inhibition may affect other cells in the retina, which could support cone survival in the absence of rods [46, 47]. A single intravitreal injection of HDAC inhibitor fully protected cones for one week and significantly slowed down cone loss up to 90 days even when all rods had already degenerated. Interestingly, the *rd1*^*TN-XL*^ cone loss followed exponential kinetics, as suggested for other forms of neurodegeneration [48]. The observed exponential cone loss in the *rd1*^*TN-XL*^ retina excludes the possibility of the cumulative damage caused by the loss of rods and suggests that secondary dying cones can be rescued at any time, although with the disease progression, fewer cells will be amenable for rescue. Therefore, a treatment even at late stages of the disease is likely to be beneficial [48]. Indeed, our data on HDAC-induced cone protection at different stages of *rd1*^*TN-XL*^ cone loss (PN19 and PN21) confirm this and is furthermore in line with other observations indicating that the window-of-opportunity for the treatment of retinal dystrophies is much broader than it is currently considered [2, 49].

Preserving retinal function in the absence of rods is the ultimate goal of neuroprotective therapies for late-stage RP. Recent studies suggest that therapies specifically targeting cones at stages where most of the rods are still present could preserve visual function in RP mouse models [3, 10]. Here we have demonstrated an intact cone-driven retinal circuitry in the TSA-treated *rd1*^*TN-XL*^ retinal explants, even in the absence of rods, by recording cone-mediated light-induced responses from large populations of individual RGCs. Unfortunately, the low cone number and the impaired morphology of the remaining cones, at the stages where rods have already fully degenerated, is a major obstacle in assessing visual improvements by standard functional tests like ERG [50]. While we did not detect changes in ERG-wave amplitudes in *rd1*^*TN-XL*^ mice treated at such late stages of degeneration, a modest improvement of ERG recordings was detected in *rd10* mice following the treatment. Notably, despite ERG amplitudes remaining lower than in wild-type mice, the significant improvement in visually-driven behavior suggests that the potential benefits for the daily tasks of RP patients may be achievable even at the late stages of the disease [51]. It is very challenging to assess the effectiveness of vision therapy, even in patients. For example, no improvements in ERG recordings were reported in patients following RPE65 gene augmentation therapy, although the useful vision was significantly increased in some patients [52]. Even in the patient who showed a dramatic improvement in the obstacle-path mobility test, *i.e*., functional vision, the ERG did not differ significantly from baseline. Therefore, it is expected that even a minor but significant improvement in visual function by the ERG should be positively correlated with treatment outcome.

For the treatment of retinal diseases, intravitreal injections are the preferred route of administration, as larger volumes of a drug can be applied, and the risk of retinal damage is reduced. However, due to an extensive retinal circulation and the brain-retinal-barrier’s permeability for low molecular weight compounds, intravitreally injected TSA is cleared from the mouse eye in less than 2 h [14]. Still, a single injection led to a long-lasting cone survival, suggesting the possibility of epigenetically induced pro-survival mechanisms that could counteract environmental insults caused by the absence of rods. While HDAC inhibition induces changes in gene expression within minutes [17], the effects of transcriptional regulation can persist for months [53].

In advanced RP, cones are exposed to high oxidative stress and inflammation [3, 4, 54] that elicit a broad range of responses, from proliferation to cell death [55]. Our RNA-seq data suggested changes in major pathways involved in regulating the cellular response to oxidative stress, the mitogen-activated protein kinases (MAPK), and phosphoinositide 3-kinase activated protein kinase (PI3K-Akt) pathways. While previous studies linked the neuroprotective effect of docosahexaenoic acid [56] and leukemia inhibitory factor to MAPK pathway activation in rod photoreceptors [57], our study highlights the role of MAPK signaling in the prevention of secondary cone degeneration. TSA-induced cone survival was significantly reduced by U0126, linking cone survival in secondary cone degeneration to the ERK pathway [39]. A similar reduction in cone survival was also observed when PI3K signaling was inhibited by LY294002, highlighting the importance of the PI3K-Akt pathway also in cone photoreceptor survival [58]. While more specific studies are necessary to precisely address the protective role of MAPK and PI3K-Akt activation in secondary dying cones, these results not only validate our RNA-seq data but also suggest that HDAC inhibition may lead to the expression of neurotrophic factors that mediate neuroprotection *via* activation of these pathways [59, 60]. Indeed, our data suggest that an increase in transcription of neurotrophic factors, such as *Bdnf*, may contribute to cone survival. Although BDNF-induced neuroprotective effects on light- or mutation-induced photoreceptor degeneration was recognized as early as 1992 by LaVail et al. [61], our study indicates a direct upregulation of *Bdnf* transcription in the cones protected from secondary degeneration.

Cone starvation has also been recognized as one of the main contributors to secondary cone death in RP [1, 10, 62]. Autophagy is the primary cellular mechanism for self-nourishment and recycling of metabolites to supplement macromolecules and energy under severe starvation [42]. The beneficial role of autophagy is demonstrated for the clearance of misfolded proteins in mutation-affected photoreceptors or dysfunctional RPE in age-related macular degeneration (AMD) [43, 63]. Nevertheless, in secondary cone degeneration, it remained unclear whether increased autophagy is beneficial or not [1, 64]. The observed upregulation of *Atg5* transcription and an increased number of autophagolysosomes in TSA-treated cones are in line with the previously reported effects of HDAC inhibition on autophagy induction [65] and highlight autophagy as a protective mechanism in secondary cone degeneration.

In our study, a single intravitreal TSA injection afforded a prolonged delay in cone cell death. Nevertheless, degeneration proceeded, albeit with slowed kinetics. This could stem from rapid TSA degradation and/or possible off-target effects. TSA is one of the most well-studied hydroxamate HDAC inhibitors, reversibly inhibiting class I and II HDACs [66]. Low nanomolar doses of TSA efficiently inhibit HDAC in tumor and non-tumor cells [67], but as TSA undergoes rapid metabolic degradation [68], systemic treatments require repeated administration [67]. Translating our approach into a clinical application may benefit from the development of drug delivery systems that enable a sustained, long-term release of an HDAC inhibitor [69]. Alternatively, as a single intravitreal injection could prevent cone degeneration for one week in mice with extremely fast photoreceptor loss, monthly injections could have similar effects in patients where the loss of cones spans over decades [70]. Future studies may also address the development of more specific and effective HDAC inhibitors that may act longer on ocular tissue. Specifically, other drugs belonging to the group of hydroxamic acids, such as vorinostat, belinostat, and panobinostat, might be of interest as we show that clinically approved panobinostat protected cone photoreceptor ex vivo to a similar extent as TSA. LINCS analysis indicated that also vorinostat may be repurposed for RP treatment. Notably, the clinical data already available [71] would greatly facilitate the repurposing for RP treatment, while the side-effect profile associated with systemic application in cancer therapy would likely be of lesser concern if these drugs were used only locally in the eye.

Our previous studies showed HDAC overactivation at the peak of photoreceptor loss in ten rodent models for retinal dystrophies [15, 35]. Aberrant HDAC activity was detected in photoreceptors regardless of whether underlying mutations affect the phototransduction cascade (*rd1*, *rd10*, *Rho* KO, S334ter, P23H, *Cngb1* KO, *Cnga3* KO, *cpfl1*), the photoreceptor outer segment structure (*rd2*) or the visual cycle (*Rpe65* KO). This suggests that increased HDAC activity may contribute to the mutation-related photoreceptor cell death caused by cGMP accumulation [69], endoplasmic reticulum stress [72, 73], or microglia activation [74]. Importantly, HDAC inhibition is also extensively discussed as a therapeutic option for other diseases such as cancer [75], Alzheimer’s disease [76], or Duchenne muscular dystrophy [77]. Consequently, in the view of the use of HDAC inhibition for the treatment of various diseases, and our previous data linking HDAC overactivation to rod loss in different mouse models of RP, this study highlights HDACs as a common denominator of both mutation-induced rod cell death and secondary cone degeneration and provides a unique therapeutic option for the treatment of RP regardless of the stage of degeneration.

Finally, detrimental environmental conditions inducing cone loss at the late stages of RP share essential similarities with AMD, the leading cause of blindness in the industrialized world [78]. Although RPE cells are considered the primary target for AMD pathology, loss of cone photoreceptors in the macular region in AMD patients characterizes the final stages of the disease [79]. Since RPE dysfunction in AMD may expose cones to a milieu similar to the one in late-stage RP, HDAC inhibition holds significant promise also for the treatment of more common forms of retinal degeneration.

## Supplementary information

Supporting info

Figure S1.

Figure S2.

Figure S3.

Figure S4.

Figure S5.

Figure S6.

Figure S7.

Figure S8.

Supplementary Table 1.

## Data Availability

All analyzed datasets are included in the manuscript and SI Appendix.
