# Supplementary material for: HDAC inhibition ameliorates cone survival in retinitis pigmentosa mice
Source: Cell Death Differ. 2020 Nov 6;28(4):1317–32. doi: 10.1038/s41418-020-00653-3 (PMC8026998; doi:10.1038/s41418-020-00653-3)
Supplement: Supplementary file 1 — Supporting info [file 41418_2020_653_MOESM1_ESM.docx]

**Supplementary information for**

HDAC inhibition ameliorates cone survival in retinitis pigmentosa mice

Marijana Samardzija, Andrea Corna, Raquel Gomez-Sintes, Mohamed Ali Jarboui, Angela Armento, Jerome E. Roger, Eleni Petridou, Wadood Haq, Francois Paquet-Durand, Eberhart Zrenner, Pedro de la Villa, Günther Zeck, Christian Grimm, Patricia Boya, Marius Ueffing and Dragana Trifunović

Dragana Trifunović

Email: dragana.trifunovic@uni-tuebingen.de

Figure S1. Quantification of cone photoreceptors. (A) Representative image of a PN30 flat-mount *rd1^TN-XL^* retina, with TN-XL biosensor labeling cone cell bodies, segments, and axons. In the *rd1^TN-XL^* retina at PN30 most rods have degenerated, leading to almost horizontal positioning of remaining cone photoreceptors due to the lack of structural support from rods. (B) An example of a retinal cross-section from an *rd1^TN-XL^* mouse at the same age. DAPI (grey) facilitated the distinction of cell bodies (filled arrows) from segments marked with arrows. Only cone cell bodies with clearly visible nuclei were counted. (C) To account for center to periphery gradient of cone loss present in the *rd1^TN-XL^* retina, cones were quantified, per 100 µm of ONL length, at two positions in the central retina (ventral and dorsal, corresponding to -10° and 10° of eccentricity from the ON, respectively) and around -80° and 80° covering far periphery. (D) Spider diagrams showing the number of cones at indicated positions in the retina following TSA or sham treatment at P19 and analyzed as indicated. PN26 (n=5 mice), PN30 (n=7), PN37 (n=5), PN45 (n=9), PN60 (n=8) and PN90 (n=6). Statistical significance for each time point was assessed using two-way ANOVA, with * *p < 0.05*, ** *p < 0.01*, *** *p < 0.001, p < 0.0001*. Scale bars: 50 µm. ON, optic nerve.

**Figure S2.** **Expression of TN-XL biosensor in cone photoreceptors.** Cross-sections of sham-injected (A; control) and TSA-treated (B) *rd1^TN-XL^* retinas immunostained for cone arrestin (CAR) at PN26, 30, 37, 45, 60, and 90. Scale bars: 20 µm. **Figure S3.** ***TSA slows down rd1^TN-XL^ cone cells death in vivo.*** The Hedges' *g* for comparisons of the cone survival ratio (PN30-90) against the PN26 ratio are shown in the Cumming estimation plot. The raw data are plotted on the upper axes. On the lower axes, the mean differences are plotted as bootstrap sampling distributions. Each mean difference is represented as a dot. Each 95% confidence interval (CI) is indicated by the ends of the vertical error bars. The effect sizes and CIs are reported below as effect size [CI width lower bound, upper bound, *p* value of the two-sided permutation t-test]. The unpaired Hedges' *g* between PN26 and PN30 is -0.346 [95.0%CI -1.53, 0.917, *p=0.51*]; PN26 and PN37: 0.442 [95.0%CI -0.909, 2.05, *p=0.464*]; PN26 and PN45: 0.358 [95.0%CI -1.2, 1.4, *p=0.511*]; PN26 and PN60: 0.754 [95.0%CI -0.482, 1.79, *p=0.184*]; PN26 and PN90: 1.41 [95.0%CI 0.624, 2.69, **p=0.039*].

**Figure S4.** **Panobinostat protects *rd1^TN-XL^* cones *ex vivo*.** Retinal explants of *rd1^TN-XL^* mice isolated at PN19 were treated with a clinically approved HDAC inhibitor, Panobinostat, for 7 days. (A) Representative images of retinal cross-sections used to determine cone numbers (B). As compared to controls, more cones were detected in Panobinostat-treated mice (5.23 ± 0.69 *vs*. 7.8 ± 1.35, respectively). n=4 mice, *p=0.0268,* unpaired, two-tailed t-test. ONL, outer nuclear layer; INL, inner nuclear layer; GCL, ganglion cell layer. Scale bars: 50 µm.

**Figure S5.** **Flow cytometry sorting of TN-XL labeled cone photoreceptors.** (A) FACS plots for a control *rd1^TN-XL^* PN19-26 retinal explant. TN-XL positive cone photoreceptors were gated for: singlets forward scatter (FSC-A *vs.* FSC-H) / singlets side scatter (SSC-A *vs.* SSC-H) / viable cells (FSC-A *vs.* SSC-A) / TN-XL cells (FSC-A *vs*. TN-XL-A, magenta). (B) Plots from a TSA-treated explant. Percentages of fluorescently labeled cones are highlighted in green. (C) The purity of sorted TN-XL positive cells was determined by performing post-sort FACS analysis on a resorted control and treated (D) sample.

**Figure S6.** **Validation of differentially expressed genes following the TSA treatment.** qRT-PCR validation of the differential expression of selected genes within the MAPK/PI3K-Akt pathway (*Igf1*, *Fgf9,* and *Trkβ*), autophagy (*Beclin1*), and glycolysis/mTOR (*Ldha*) pathways in FACS-sorted cones. Fold changes are shown relative to corresponding controls. Data are shown as mean ± SEM (n = 4 animals). Mann-Whitney nonparametric test was used to assess the statistical difference. *n.s*.- non-significant.

**Figure S7. Autophagy after HDAC inhibition *ex vivo*.** (A) Representative images of a single confocal plane of retinal cross-sections from the *rd1^TN-XL^* retina explanted at PN19 and treated *ex vivo* for 7 days with control or TSA-medium. Arrows label colocalization between autophagosomes stained with LC3 in cyan and lysosomes labeled with LAMP1 in red. (B) Quantification of the number of colocalized puncta per cone in 4 sections obtained from 2 animals per condition. Data are shown as mean ± SEM. Numerical p-values by Mann-Whitney nonparametric test. Scale bar 10 μm. ONL, outer nuclear layer; INL, inner nuclear layer.

**Figure S8.** **LINCS** **Query.** The web-based tool used for identification of small molecules mimicking and reversing the gene expression changes in TSA-protected cones confirmed the specificity of the observed changes to HDAC inhibitors, trichostatin A (TSA) and vorinostat.
