# Supplementary figures and images for: HDAC inhibition ameliorates cone survival in retinitis pigmentosa mice
Source: Cell Death Differ. 2020 Nov 6;28(4):1317–32. doi: 10.1038/s41418-020-00653-3 (PMC8026998; doi:10.1038/s41418-020-00653-3)

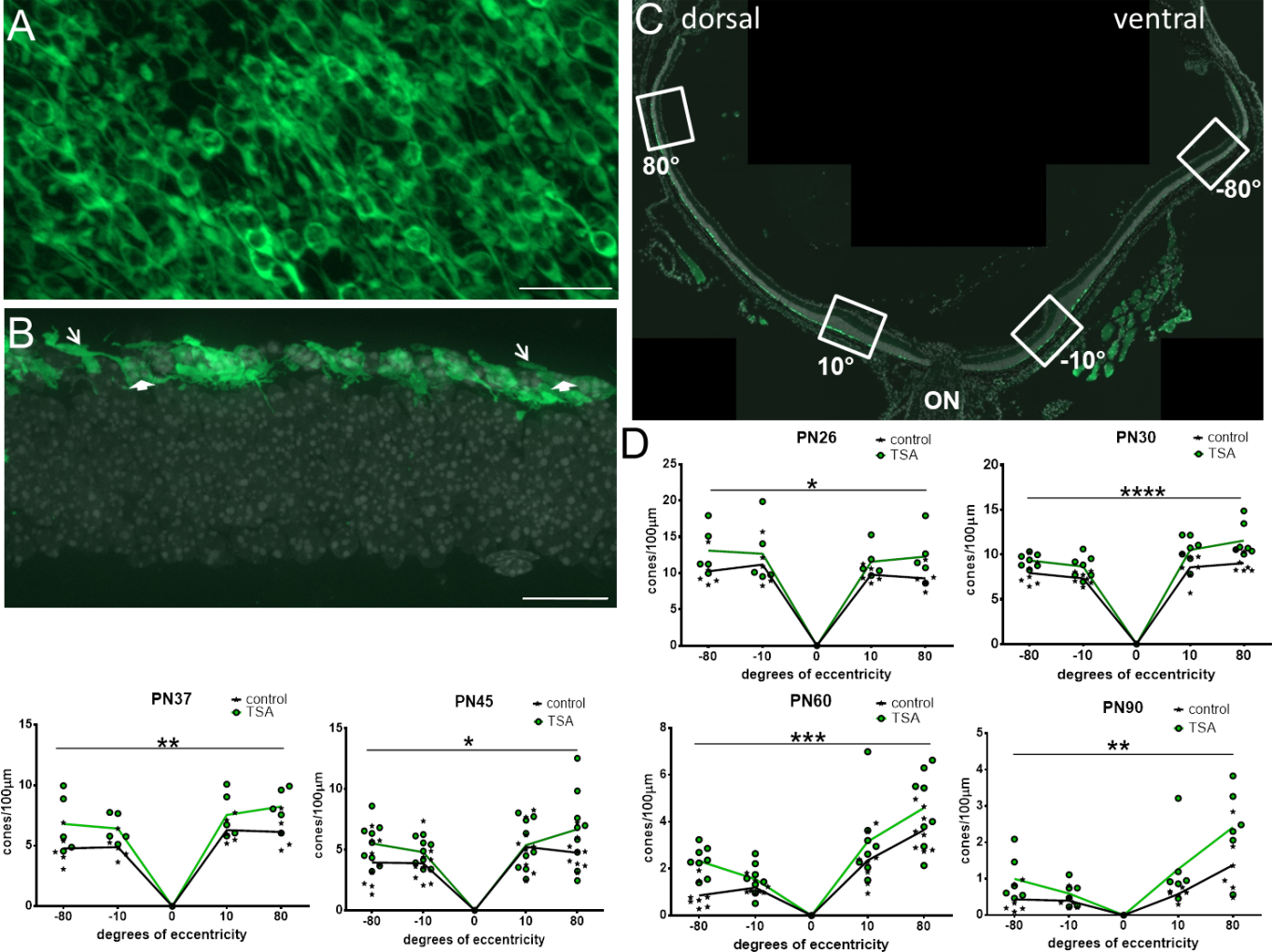

Supplement: Supplementary file 2 — Figure S1. [file 41418_2020_653_MOESM2_ESM.png]

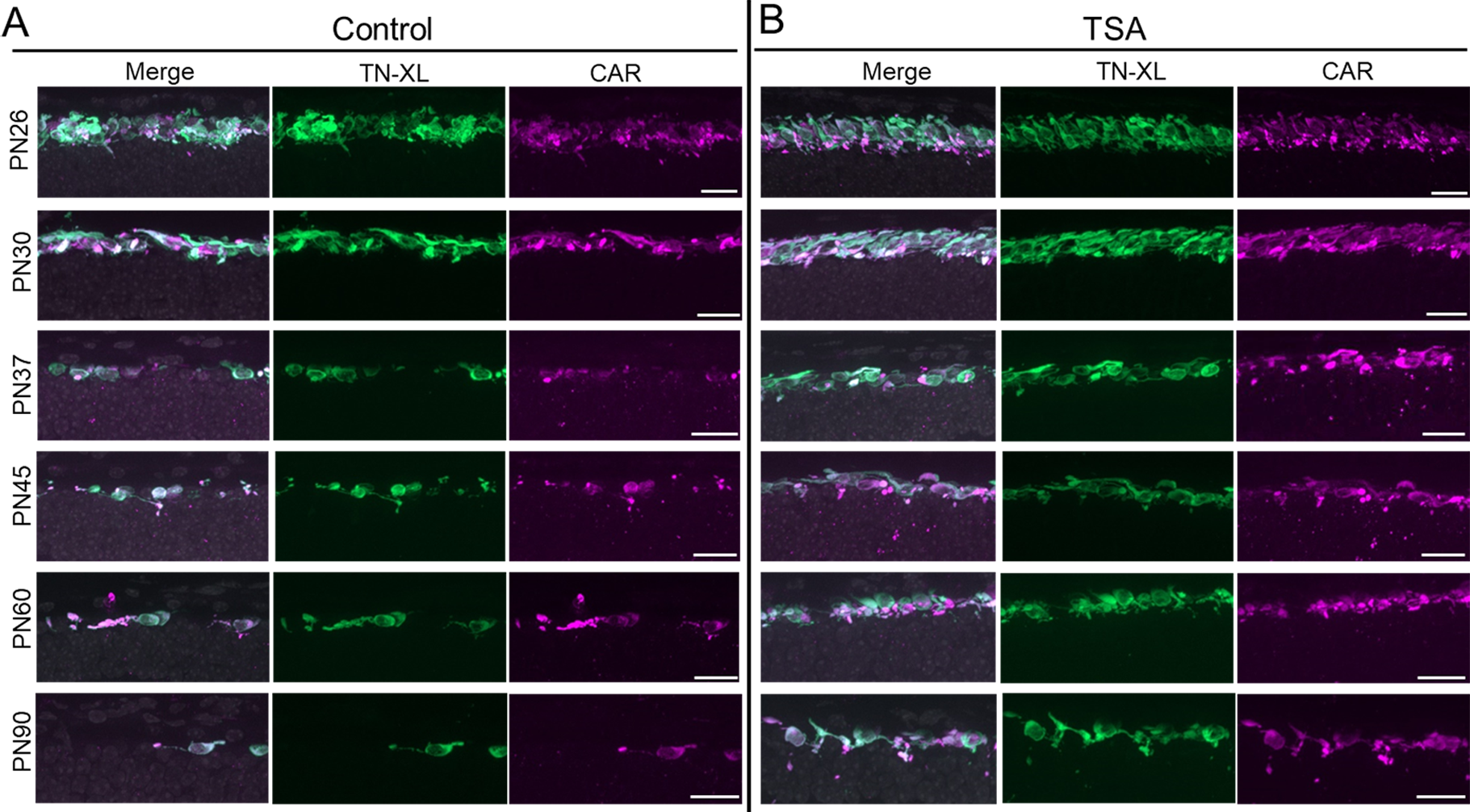

Supplement: Supplementary file 3 — Figure S2. [file 41418_2020_653_MOESM3_ESM.png]

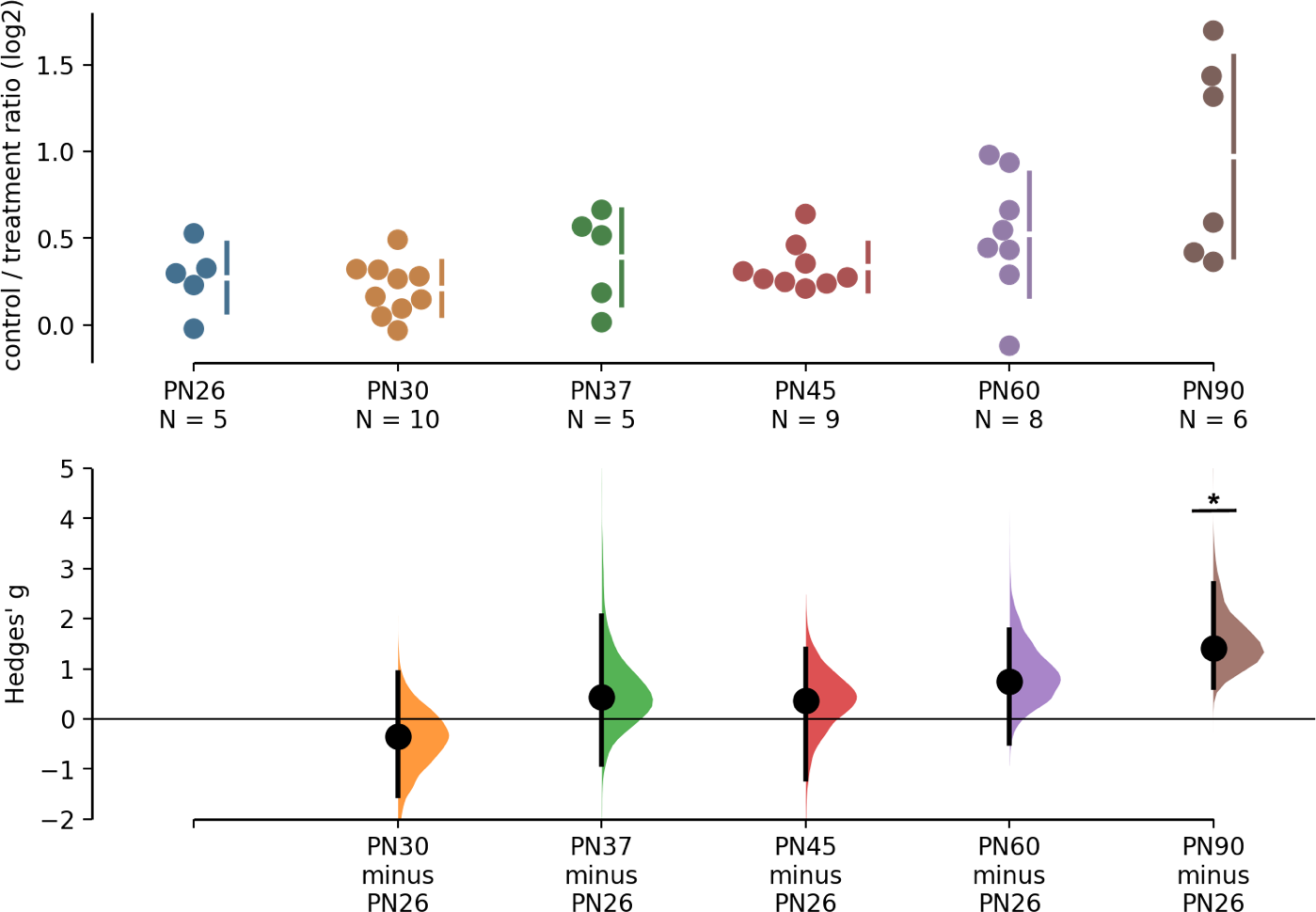

Supplement: Supplementary file 4 — Figure S3. [file 41418_2020_653_MOESM4_ESM.png]

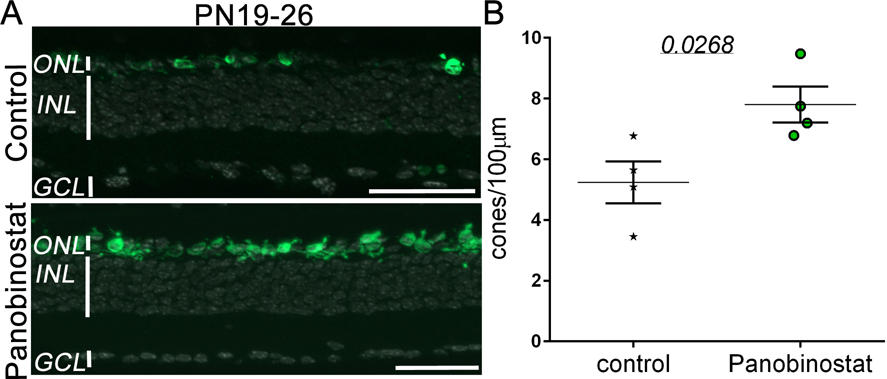

Supplement: Supplementary file 5 — Figure S4. [file 41418_2020_653_MOESM5_ESM.png]

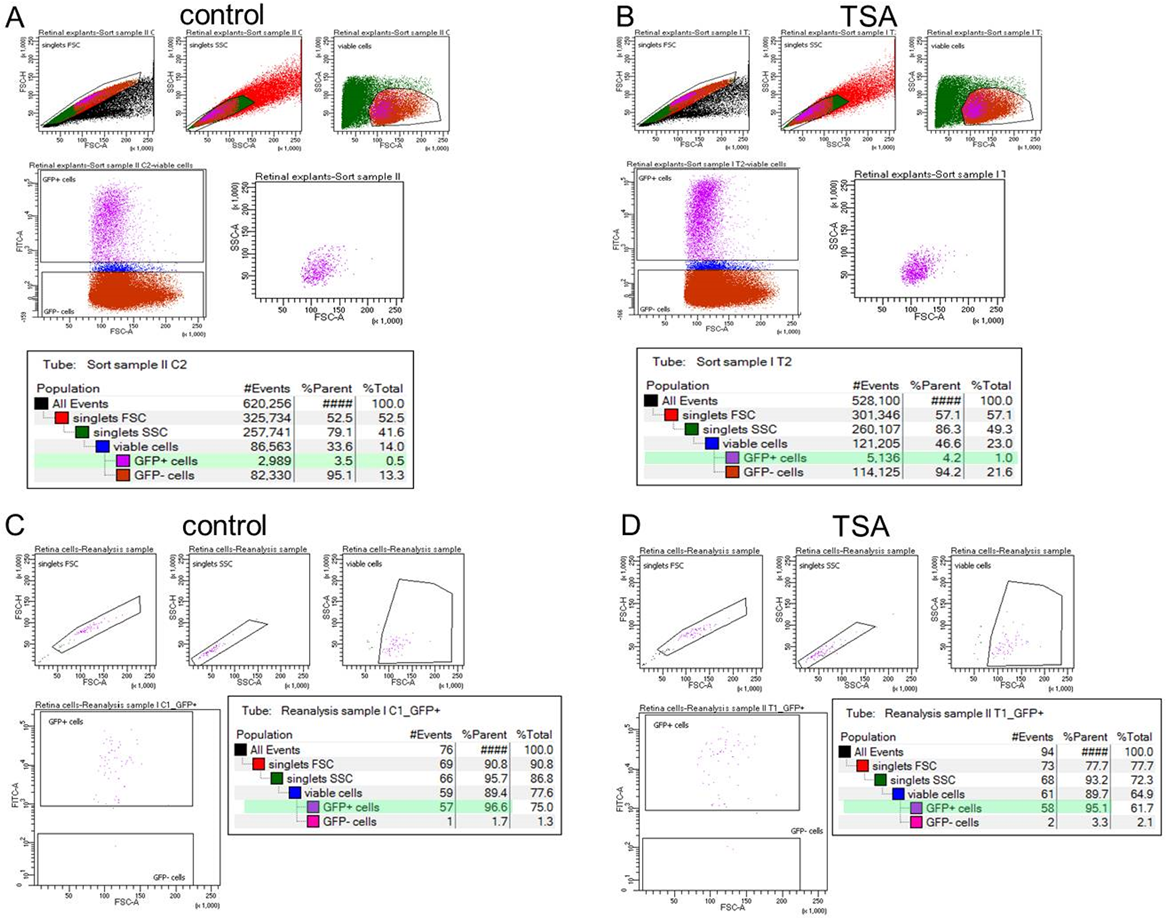

Supplement: Supplementary file 6 — Figure S5. [file 41418_2020_653_MOESM6_ESM.png]

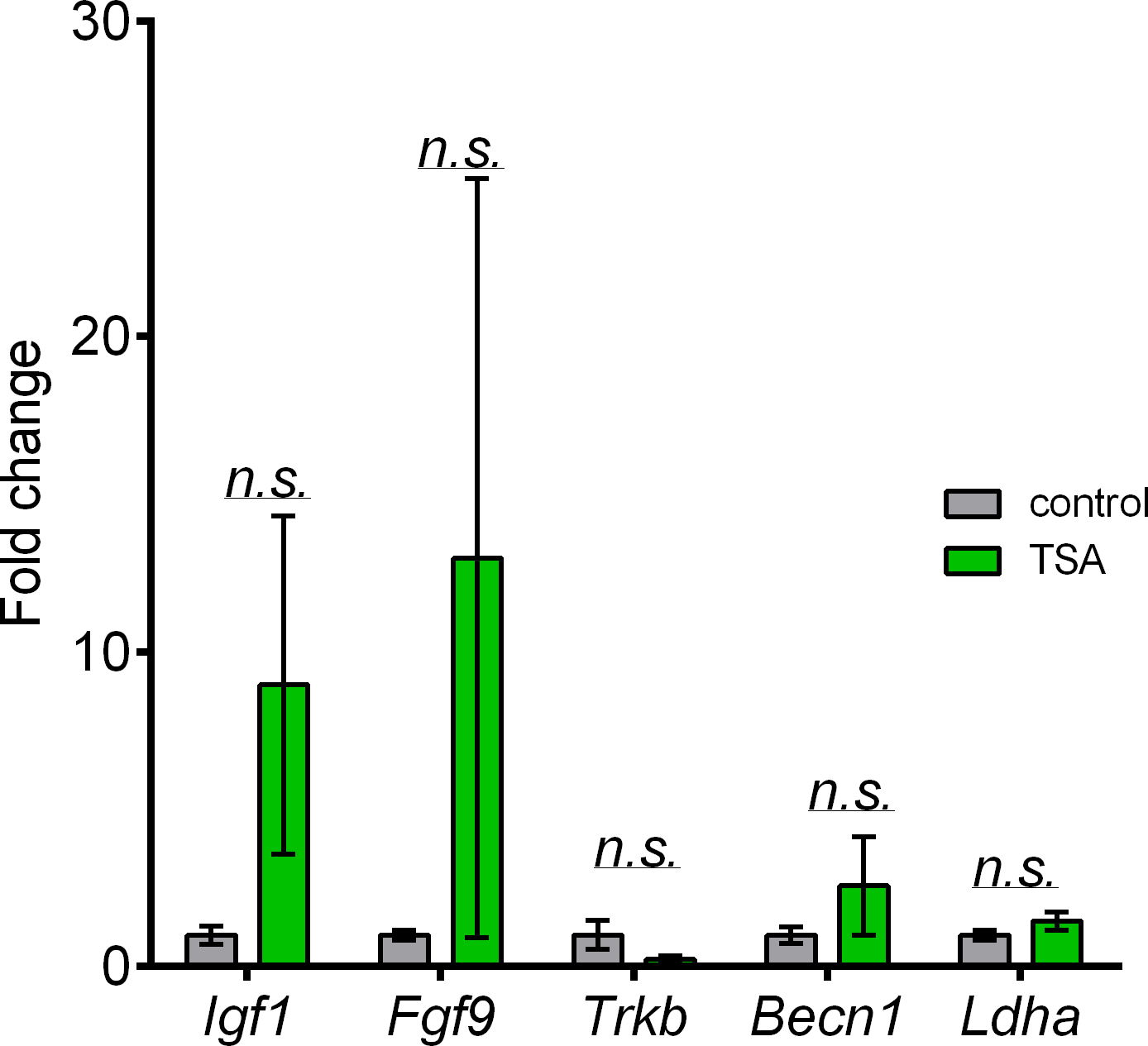

Supplement: Supplementary file 7 — Figure S6. [file 41418_2020_653_MOESM7_ESM.png]

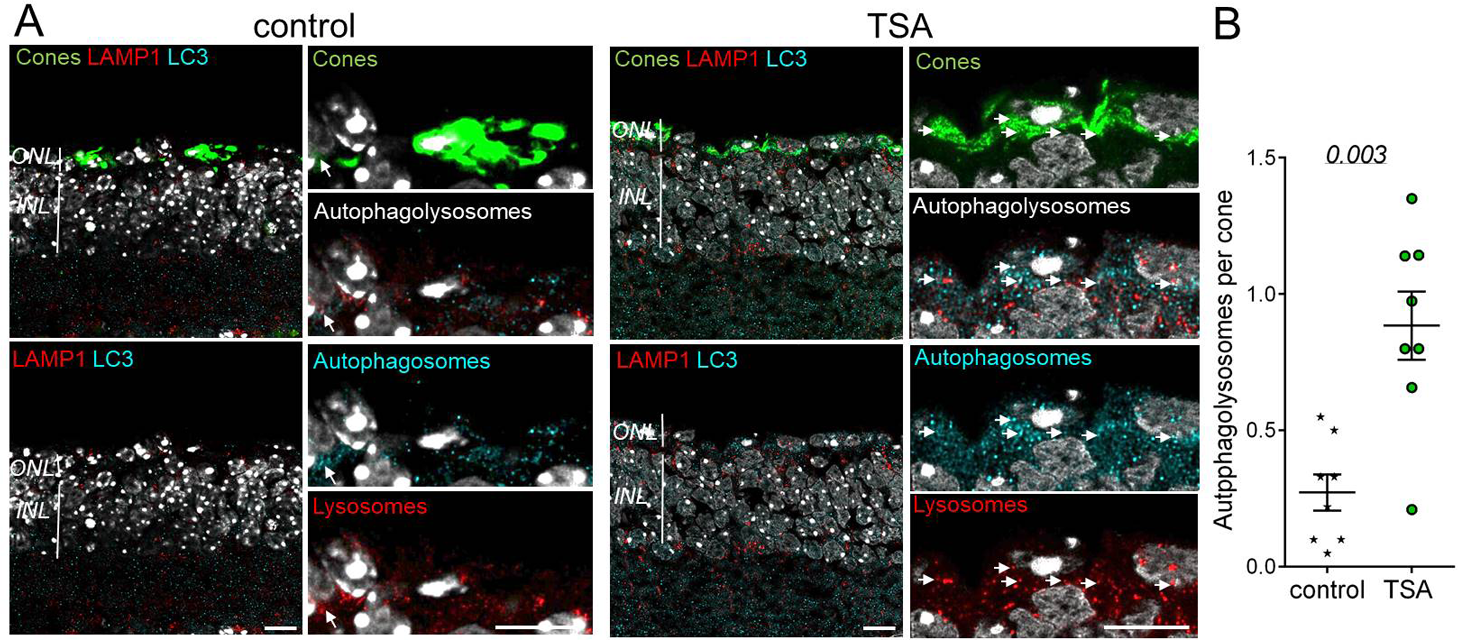

Supplement: Supplementary file 8 — Figure S7. [file 41418_2020_653_MOESM8_ESM.png]

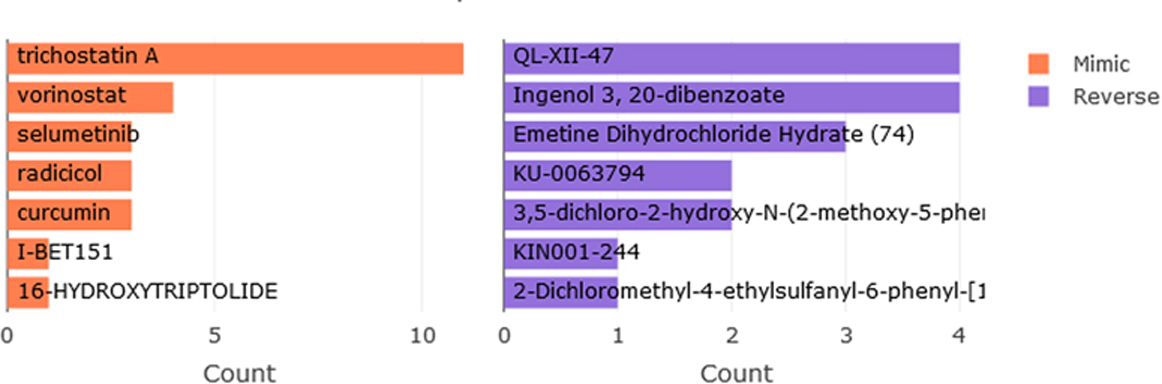

Supplement: Supplementary file 9 — Figure S8. [file 41418_2020_653_MOESM9_ESM.png]
