# Supplementary material for: HDAC inhibition ameliorates cone survival in retinitis pigmentosa mice
Source: Cell Death Differ. 2020 Nov 6;28(4):1317–32. doi: 10.1038/s41418-020-00653-3 (PMC8026998; doi:10.1038/s41418-020-00653-3)
Supplement: Supplementary file 10 — Supplementary Table 1. [file 41418_2020_653_MOESM10_ESM.docx]

Table S1. **Primers used for qRT-PCR analysis.**

| **Gene** | **Forward primer (5’-3’)** | **Reverse primer (5’-3’)** |
| --- | --- | --- |
| *Bdnf* | TTCGGCCCAACGAAGAAA | TCCTCCAGCAGAAAGAGTAGA |
| *Atg5* | CAAGCCAAGGAGGAGAAGATT | TGCATTTCACGAGAAGAGGAG |
| *Gapdh* | GGAGAAACCTGCCAAGTATGA | TCCTCAGTGTAGCCCAAGA |
| *Igf1* | TCCAGTTGCTCTAAGTTTCTCTC | CGTGGGAAGAGGTGAAGATAAG |
| *Fgf9* | ATCAGGTTCAGTTCCTGCTTTA | CGAGAGAGTGGTTTGGCTATC |
| *Beclin1* | CAGGAACTCACAGCTCCATTAC | CCATCCTGGCGAGTTTCAATA |
| *Trkß* | GGAGCATTCCATTCCAGGTT | CTCTCTTCTCCCTCTCCTTTCT |
| *Ldha* | AGGCTCCCCAGAACAAGATT | GTCAACAAGGGCAAGCTCAT |
